# The Local Parastomal Hernia (LoPa) Repair: A Novel Approach to Parastomal Hernia Repair

**DOI:** 10.3389/jaws.2025.15878

**Published:** 2026-01-14

**Authors:** Ali Al Mukhtar, Agneta Montgomery, Kristin Johnson, Peder Rogmark, Stefan Öberg, Tomas Vedin, Ulf Petersson, Marie-Louise Lydrup

**Affiliations:** 1 Department of Clinical Sciences, Faculty of Medicine, Lund University, Malmö, Sweden; 2 Department of Surgery, Skåne University Hospital, Malmö, Sweden; 3 Department of Translational Medicine, Radiology Diagnostics, Lund University, Malmö, Sweden; 4 Department of Imaging and Physiology, Skåne University Hospital, Malmö, Sweden; 5 Department of Clinical Sciences, Faculty of Medicine, Lund University, Lund, Sweden; 6 Department of Surgery, Helsingborg Hospital, Helsingborg, Sweden

**Keywords:** hernia, mesh, parastomal hernia, parastomal hernia repair, technique

## Abstract

**Purpose:**

Surgical repair of parastomal hernias (PH) is challenging, mainly due to high recurrence rates. The Local Parastomal repair (LoPa) is a novel technique utilizing a retromuscular synthetic mesh with an outward-facing collar. This study describes the LoPa technique and evaluates its outcomes.

**Methods:**

This single-centre study retrospectively reviewed 39 consecutive patients who underwent LoPa repair for a PH between 2017 and 2021. Long-term follow-up, including physical examination and quality of life assessment, was conducted. The primary outcome was PH recurrence diagnosed clinically or by CT scan.

**Results:**

For the 39 patients included, the mean age and BMI were 71 years and 27 kg/m^2^, respectively. The most common ASA score was III (48.7%). The median length of stay was 3 days with no Clavien-Dindo ≥4 complications observed. At a median follow-up of 47 months, the overall recurrence rate was 33.3% (12/36 patients). Postoperative general health status was comparable to the Swedish general population, though recurrence was associated with more pain and anxiety.

**Conclusion:**

The LoPa technique is a safe and feasible PH repair, offering low short-term morbidity and a short length of stay. It is an option for repairing isolated PH, especially in patients with comorbidities. While the 33.3% recurrence rate is a concern, it is comparable to other techniques with similar follow-up. These preliminary findings warrant validation in larger prospective trials.

## Introduction

Parastomal hernia (PH) is a common long-term complication after stoma creation with reported incidences as high as 81% [1–4]. Among patients operated for colorectal cancer in Sweden, nearly 20% end up with a permanent stoma and PH thereby constitutes a significant clinical concern [5].

PH can have a major impact on the patient’s quality of life [6–9], with symptoms such as abdominal pain, deformity, leakage and skin irritation due to difficulties fitting stomal appliances. It can also lead to incarceration and potentially life-threatening bowel strangulation requiring emergency surgery.

Surgical options for treating PHs include stoma reversal, stoma relocation, and PH defect repair. Stoma reversal is only possible in a small number of patients, and relocation has inherent risks of developing incisional hernias at the former stoma site and PH at the new site [10, 11]. The use of surgical mesh is considered the gold standard for a durable repair, as suture repair is associated with a higher risk of recurrence and surgical site infection (SSI) [2, 12, 13].

The surgical repair of PH remains a significant clinical challenge. No single technique is universally accepted and reported recurrence rates vary from 0% to 90% [2, 13–22]. Comparative studies assessing the efficacy of different repair techniques are scarce [2]. The most commonly applied mesh repair approaches, the keyhole and Sugarbaker techniques [13], typically require entry into the abdominal cavity via laparotomy or laparoscopy, followed by adhesiolysis prior to mesh repair [23]. This entails a non-negligible surgical trauma and risk of complications. Underscoring the challenge of PH repair a recent randomized clinical trial comparing open retromuscular Sugarbaker vs. keyhole repairs, performed by experts in advanced abdominal wall reconstruction, showed recurrence rates of 17% and 24%, respectively, at 2 years follow-up [22].

Ideally, PH repair would involve minimal surgical trauma with low risk of complications and low recurrence rate. The Local Parastomal hernia repair (LoPa) technique was developed at the Abdominal Wall Surgery unit in Malmö, Sweden, with the intention to provide a durable mesh repair while minimizing surgical trauma for patients with PH without a concomitant incisional hernia requiring repair.

In this article, the LoPa repair technique is described and the outcomes in patients treated with this technique under a 5-year period are reported.

## Materials and Methods

### Study Population and Data Collection

All patients with a symptomatic PH, 18 years or older, who underwent a LoPa repair at Skåne University Hospital in Malmö, Sweden between 1 January 2017 and 31 December 2021 were included in this retrospective study. Patients were identified via International Classification of Disease (ICD-10) diagnosis codes for parastomal hernias (K43.3, K43.4, and K43.5), and the Swedish procedure coding classification (KVÅ) codes, recorded in the regional patient administrative system and local operation registration software.

Medical records were reviewed for patient demographics and operative details. To minimize the risk of information bias during data collection, the guidelines for retrospective medical record reviews as outlined by Vassar and Holzman [24] were followed. A proforma protocol detailing the collection process of the parameters was created, tested for applicability, and revised accordingly. The data collection process was overseen by a single designated collector, and any uncertainties in data interpretation were discussed within the research team before recording.

### Definitions and Radiological Assessment

A PH was defined according to the European Hernia Society classification [25] as “an abnormal protrusion of the contents of the abdominal cavity through the abdominal wall defect created during placement of a colostomy, ileostomy or ileal conduit stoma.” The size of the hernia defect was measured on CT scans, as operative measurements were not always documented in the surgical notes. The available CT scans were performed for various indications with different protocols, i.e., with or without intravenous contrast, not specific for hernia diagnosis. CT scans were independently reviewed by one radiologist and one abdominal wall surgeon, neither of whom was involved in the perioperative care of patients. To distinguish mesentery fat belonging to the stoma limb, in case of a siphon which is not considered a hernia, from protrusion of omental fat representing an abnormal protrusion and thereby a hernia, is not always simple. To do so, the examiners followed the course of the vessels and if this led to the mesenteric vessels the fat was considered stoma-related and not a hernia. If the course was for omental vessels and heading towards the transverse colon it was classified as a hernia.

The degree of contamination in the surgical field was classified according to the Centres for Disease Control and Prevention (CDC) [26]. Short-term complications were defined as occurring within 90 days postoperatively and long-term complications as those occurring later. Mandatory discharge criteria were passage of faeces/urine through a viable stoma. Surgical site infection (SSI) was categorized according to CDC classification [27] as superficial, deep, or organ space. Surgical site occurrences (SSO) were defined as seroma, hematoma, mucocutaneous separation, and enterocutaneous fistula formation. Additionally, surgical complications were categorized according to the Clavien Dindo classification [28].

### Long Term Follow-Up and QoL Assessment

The long-term follow-up consisted of physical examinations by one of two designated abdominal wall surgeons and completion of QoL questionnaires. The primary outcome was PH recurrence detected during follow-up visits or on available CT scans performed after the repair. Secondary outcomes were operative time, length of stay (LOS), SSI and SSO, postoperative stoma-related complications, reoperation rate and QoL assessment during follow-up.

QoL questionnaires were completed, either in person or via telephone interview. Two validated instruments were used: the generic EQ-5D-5L for overall health status [29], and the Colostomy Impact Score, specifically designed to assess the QoL of patients with permanent colostomies [30]. The use of EQ-5D-5L was approved for research use by the EuroQol group (Rotterdam, Netherlands), ID: 79292. As the cohort included patients with various stoma types (colostomies, ileostomies, and ileal conduits) and since specific validated QoL instruments for non-colostomy stomas are lacking, a pragmatic approach was required, implying that all patients were assessed using the Colostomy Impact Score, with the item on faecal consistency excluded for those with ileostomies or ileal conduits to ensure the results were not misleading. Since preoperative QoL data were unavailable, results were contextualized using reference populations. EQ-5D-5L results were compared to values from the Swedish general population, and the Colostomy Impact Score to, a Swedish subgroup in a large European cohort of long-term rectal cancer survivors with stomas [31, 32]. Within our cohort, QoL for patients with a recurrence after LoPa repair was compared to patients without, and to reference values in the Swedish population. A difference greater than 10 percentage points was considered clinically meaningful.

### Statistics

Categorical variables were presented as counts and percentages. Normally distributed data were reported as mean and standard deviation (SD), while non-normally distributed data were presented as median and interquartile range (IQR).

### Operative Description of the LoPa Technique

LoPa repair is performed under general anaesthesia. Intravenous antibiotic prophylaxis is administered preoperatively. The first step of the procedure is to close the mucosa of the stoma using a running suture to prevent faecal spillage which is followed by sterile preparation and draping. A skin incision is made close to and around the stoma, followed by dissection of the stoma from the skin and subcutaneous tissue.

**FIGURE 1:**
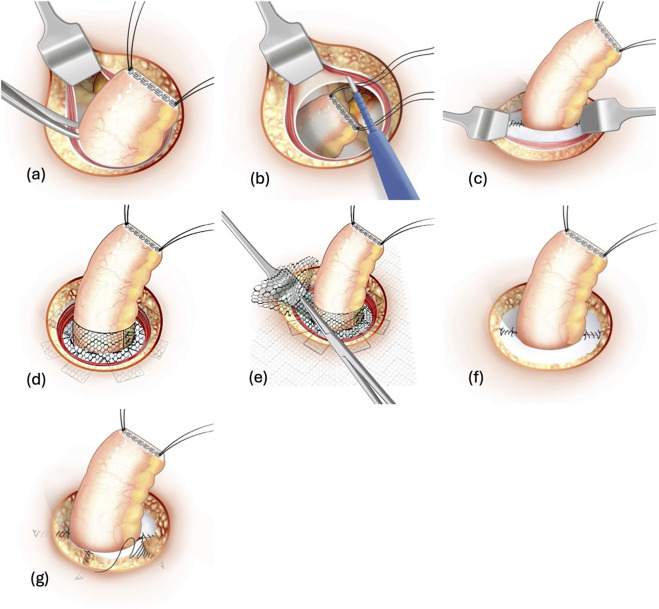
Illustration of the LoPa technique. The key steps are shown: **(a)** Dissection of the stoma from the abdominal wall. **(b)** Developing the retromuscular space. **(c)** Closure of the posterior rectus fascia. **(d)** Mesh collar fitted around the stoma. **(e)** Flat mesh placed around the mesh collar and anchored onto the posterior rectus fascia. **(f)** Closure of the anterior rectus fascia. **(g)** Adaptation of the subcutaneous fat around the stoma.

The hernia sac is identified and dissected down to the anterior rectus fascia. After opening the hernia sac, adhesiolysis between the sac, stoma bowel and herniated content is performed to enable hernia content reduction and to obtain adequate stomal length. When the stoma bowel has been adequately dissected, the distal part with the sutured mucosa is removed with a stapler. Long sutures are placed at the staple line to facilitate finding the stoma bowel at the end of the procedure, whereafter the bowel is placed intraabdominally Figure 1a.

The remainder of the hernia sac is completely dissected from the subcutaneous fat and extirpated. By incision of the anterior rectus fascia (ARF) at the stoma orifice, a retromuscular space is created between the rectus muscle and the posterior rectus fascia (PRF) or peritoneum if dissection is needed below the arcuate line Figure 1b. The PRF is sutured with a 2-0 non-absorbable monofilament polypropylene suture leaving a centralized opening in the PRF that permits passage of the stoma bowel alone Figure 1c. The retromuscular dissection should be wide enough to harbour a mesh that overlaps the re-sutured posterior fascia orifice by at least 3 cm in all directions. If necessary, a limited posterior component separation and transverse abdominis muscle release [33] can be performed, even within the confined surgical space of the stoma cavity, to achieve adequate lateral mesh overlap.

An outward-facing funnel-shaped synthetic mesh collar is custom-made by cutting a 3.5 × 15 cm strip from a 15 × 20 cm polyvinylidene difluoride (PVDF) mesh (Dynamesh CICAT®, FEG Textiltechnik GmbH, Aachen, Germany). One-centimetre cuts are made with 1-cm intervals along one of the mesh strip long sides, creating 1 × 1 centimetre mesh flaps and a 2.5 cm long collar. The flaps of the mesh strip are attached to the PRF with interrupted 2-0 polypropylene sutures around the edge of the PRF orifice. The stoma bowel is brought out through the orifice and the attached collar, making sure not to twist it. The mesh collar is finally cut to fit around the stoma bowel, and the collar-edges are fixed using 2-0 polypropylene sutures Figure 1d, without suturing the collar to the stoma bowel. The remaining mesh is subsequently fashioned to fit the retromuscular dissected space, and a cruciate opening is made in the centre of the mesh which is brought down over the stoma bowel and mesh collar and placed flat onto the PRF. The mesh should extend at least 3 cm from the stoma bowel in all directions. The flat mesh is then anchored with four interrupted 2-0 polypropylene sutures at the edge of the PRF orifice Figure 1e. The collar is embedded in the rectus muscle and the ARF is sutured with 2-0 polypropylene sutures, leaving a centralized customized opening for the stoma bowel Figure 1f.

Displaced subcutaneous fatty tissue is adapted using a 3-0 slowly absorbable monofilament polydioxanone suture to create support for the overlying skin Figure 1g. The skin opening is tightened with a 3-0 polydioxanone purse string suture to adapt the opening to the size of the stoma bowel, which is finally opened and sutured with everting interrupted 4-0 polydioxanone sutures to the skin.

When using the LoPa technique for PH in ileal conduits, the same method is utilized, but after recreation of the stoma, a Foley’s catheter is inserted in the ileal conduit and kept there postoperatively for several days until stomal swelling subsides to prevent urinary retention.

For recurrent PH with prior mesh repair, and for PH developing despite the use of prophylactic mesh, the initial steps of the operation are identical to the description above. After reducing the hernia contents and excising the hernia sac, the retromuscular space is carefully developed, delineating the rectus muscle from the existing mesh which is often fused with the PRF. Subsequently the PRF is sutured with 2-0 polypropylene suture, incorporating the previous mesh, while ensuring an opening tailored solely for the passage of the stoma bowel. The stoma bowel is guided through the orifice, and a mesh collar is fitted around the stoma bowel in a similar fashion as described above, whereafter the collar flaps are sutured with a 2-0 polypropylene suture to the previous flat mesh and PRF. The subsequent phases of the procedure replicate in detail those outlined above.

## Results

A total of 56 patients underwent PH repair during the study period. Patients who had PH repair using other techniques than LoPa (n = 17) were excluded, leaving 39 patients to be evaluated.

Patient demographics and history are listed in Table 1. The mean age was 71 years, and the mean BMI 27.0 kg/m^2^. The highest proportion of patients (48.7%) had an ASA score of 3, closely followed by ASA 2 (43%). In total, more than half of the cohort was classified as ASA 3-4. A previous mesh placement near the stoma was present in 12 patients, of those 6 had recurrences after a prior PH repair, 4 had a prophylactic flat mesh placed during stoma creation, and 2 had onlay mesh from a previous fascial dehiscence.

**TABLE 1:** Patient demographic characteristics and surgical history.

Variable	Total (n = 39)
Age (year), mean (SD)	71.0 (8.0)
BMI (kg/m^2^), mean (SD)	27.0 (3)
Gender (male), n (%)	20 (51.3)
ASA, n (%)	
1	2 (5.1)
2	17 (43.6)
3	19 (48.7)
4	1 (2.6)
Smokers, n (%)	5 (12.8)
Inflammatory bowel disease, n (%)	6 (15.4)
Immunosuppressive medication, n (%)	4 (10.3)
Diabetes mellitus, n (%)	2 (5.1)
Cardiovascular disease, n (%)	16 (41.0)
Chronic obstructive pulmonary disease, n (%)	5 (12.8)
Renal insufficiency, n (%)^a^	4 (10.3)
Previous abdominal surgeries, n (%)	
1	16 (41.0)
2	14 (35.9)
3	5 (12.8)
4 or more	4 (10.3)
Stoma type	
Colostomy, n (%)	24 (61.5)
Ileostomy, n (%)	5 (12.8)
Ileal conduit, n (%)	10 (25.6)
Previous operations for ventral, incisional hernias or parastomal hernia, n (%)	
Onlay mesh repair for postoperative fascial dehiscence	2 (5.1)
Retromuscular parastomal hernia repair	5 (12.8)
Intraperitoneal sugarbaker	1 (2.6)
Previous prophylactic retromuscular parastomal mesh placement, n (%)	4 (10.3)

BMI, body mass index; ASA, american society of anaesthesiologists.

^a^ Renal insufficiency defined as GFR < 90 mL/min/1.73.

Operative details for the included patients are presented in Table 2. In one patient with a prior onlay mesh repair for fascial dehiscence, the procedure commenced as a LoPa repair, involving detachment of the ileal conduit from the PRF. However, decision was made to convert to a midline laparotomy due to extensive adhesions. After adhesiolysis, the repair was performed in accordance with the LoPa technique. Small, remote concomitant incisional hernias were present in 5 cases, 3 of which were asymptomatic and left unrepaired. Concomitant procedures were performed in 5 cases, and their duration was included in the total operative time. A preperitoneal mesh repair for small incisional hernias via separate incisions was performed in 2 patients, 2 had an open inguinal hernia repair, and 1 received a suture repair for an umbilical hernia.

**TABLE 2:** Operative details.

Variable	Total (n = 39)
Elective, n (%)	37 (94.9)
Hernia defect length (cm), mean (SD)^a^	4.0 (0.9)
Hernia defect width (cm), mean (SD)^a^	3.6 (1.1)
EHS classification of parastomal hernias, n (%)	
1	27 (69.2)
2	3 (7.7)
3	6 (15.4)
4	2 (5.1)
Classification unavailable	1 (2.6)
Conversion to midline laparotomy	1 (2.6)
Concomitant incisional hernia, n (%)	5 (12.8)
Concomitant procedures	
Incisional hernia, n (%)	2 (5.1)
Inguinal hernia, n (%)	2 (5.1)
Umbilical hernia suture repair, n (%)	1 (2.6)
CDC wound class II, n (%)	39 (100)
Operative time (min), median (IQR)	227 (170-311)

CDC, centre for disease control and prevention; EHS, european hernia society.

^a^ Hernia defect measurements were missing in one patient.

### Short-Term Outcomes

All patients attended at least one postoperative visit at the stoma nurse’s outpatient clinic within 30 days. Short term clinical outcomes are listed in Table 3. The median LOS was 3 days. Two patients were readmitted, both requiring reoperations under general anaesthesia. One patient had bowel obstruction at the level of the stoma due to kinking of the stoma bowel above the mesh. The collar part of the mesh was removed through the LoPa incision. The other patient was readmitted after 3 days with constipation and faecal impaction at the stoma level, prompting manual evacuation and irrigation under general anaesthesia. Both patients recovered postoperatively without further incidents. No Clavien-Dindo complications ≥4 were observed.

**TABLE 3:** 90-day clinical outcomes.

Variable	Total (n = 39)
Length of stay (days), median (IQR) (min, max)	3 (2–7) (1,16)
Readmission ≤30 days	
Bowel obstruction at stoma site, n (%)	1 (2.6)
Faecal impaction, n (%)	1 (2.6)
Reoperation ≤30 days, n (%)	
Bowel obstruction at stoma site, n (%)	1 (2.6)
Faecal impaction, n (%)	1 (2.6)
SSI, n^a^ (%)	
Superficial	3 (7.7)
Deep with IR^b^ assisted drainage	1 (2.6)
SSO, n^a^ (%)	
Hematoma, n (%)	3 (7.7)
Seroma, n (%)	5 (12.8)
Mucocutaneous separation, n (%)	3 (7.7)
Constipation, n^a^ (%)	5 (12.8)
Urinary tract infection, n^a^ (%)	3 (7.7)
Respiratory desaturation, n^a^ (%)	1 (2.6)
Clavien-dindo classification, n^a^ (%)	
0	24 (61.5)
1	7 (17.9)
2	5 (12.8)
3a	1 (2.6)
3b	2 (5.1)

IQR, interquartile range; SSI, surgical site infection; SSO, surgical site occurrence.

^a^ Number of patients with complications.

^b^ Interventional Radiology.

SSI was identified in 4 patients and treated with antibiotics. One was classified as deep, requiring interventional radiology-assisted drainage. One of the patients with superficial SSI experienced respiratory desaturation postoperatively due to atelectasis, which resolved with oxygen therapy, positive end-expiratory pressure and mobilization. SSOs were noted in 11 patients, none of them required procedural intervention.

### Long-Term Outcomes

**FIGURE 2:**
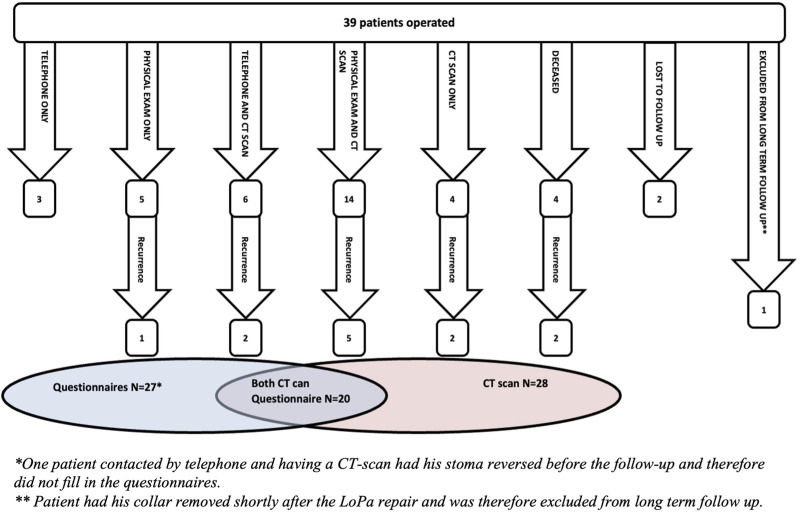
Flowchart of patient follow-up, detailing the different follow-up modalities, detected recurrences, and the overlap between available CT scans and completed QoL questionnaires.

Long-term outcomes are presented in Table 4; Figure 2. The median follow-up was 47 months. During the follow-up, 6 patients died from causes unrelated to the LoPa procedure. Of these, 2 were completely lost to follow-up, i.e., having neither postoperative CT scans nor long-term follow-up visits. The remaining 4 had CT scans performed later than 90 days postoperatively. Additionally, 4 patients declined both in-person and telephone follow-up but agreed the use of their CT-scans performed later than 90 days postoperatively. The patient who had the collar part of the mesh removed at reoperation shortly after the LoPa repair, was excluded from long-term follow-up. In total, 36 patients were evaluable with at least one of the investigational modalities.

**TABLE 4:** Long term outcomes.

Variable	Total (nr = 36)^a^
Follow up, n (%)	
CT scan only^b^	8 (22.2)
Physical exam only	5 (13.8)
Physical exam and CT scan	14 (38.8)
Telephone interview	3 (8.3)
Telephone interview and CT scan	6 (16.6)
Patients with postop CT scans, n (%)	28 (77.7)
Total follow-up (months) all investigational modalities included, median (IQR)^c^	47 (39-67)
Follow-up (months) physical exam or telephone interview, median (IQR)	54 (44-69)
Follow-up (months) CT scan only, median (IQR)	36 (22-50)
Deaths during follow-up, n (%)	6 (16.6)
BMI at follow-up (kg/m2), mean (SD)	27 (3)
Visit for stoma complication >90 days, n (%)	5 (13.8)
Surgical treatments of stoma complications during follow-up-, n (%)	
Recurrence	3 (8.3)
Stoma stricture	1 (2.7)
Total recurrence detected on CT scan and/or physical exam, n (%)	12 (33.3)
Recurrence detected on physical exam, n (%)	4 (11.1)
Recurrence on CT scan, n (%)	11 (30.5)
Recurrence by stoma type^c^	
Colostomy, n (%)	7 (31.8)
Ileostomy, n (%)	1 (25.0)
Ileal conduit, n (%)	4 (40.0)

^a^ One patient was excluded from long term follow up due to early postoperative bowel obstruction and removal of the collar. Two patients were totally lost to follow up.

^b^ Physical or telephone interview follow up was not possible in these patients.

^c^ Analysis excludes one patient with an ileostomy due to early collar removal and two patients with colostomies who were lost to follow-up. Recurrence rates are derived from the remaining 36 patients: 22 Colostomies, 4 Ileostomies, and 10 Ileal conduits.

The overall recurrence rate was 33.3% (n = 12). Of these, 11 were confirmed by CT scan while 1 was diagnosed by physical examination only. One of the CT-verified recurrences was not detected by physical examination, while all others were. Recurrences were detected at various time points: 4 within 12 months (earliest at 7 months), another 4 within 24 months, and the remaining 4 beyond 24 months (latest detection at 65 months). Regarding the three patients with small concomitant incisional hernias that were left unrepaired at the time of the LoPa procedure, none required subsequent surgical repair during the follow-up period.

Postoperative CT scans were available for 28 patients, whereof 8 were performed to investigate a suspected PH recurrence. In 4 of these cases, the scan was prompted by patient-reported symptoms, such as a visible bulge. In the other 4 cases, the scan was initiated by a physician to rule out a recurrent PH as a cause for abdominal pain, bowel obstruction, or urinary outlet issues in patients with ileal conduits.

Surgical intervention for stomal complications was required in 4 patients. Three were treated for PH recurrences: 2 underwent a local repair involving stoma detachment and mesh tightening, and the third patient, with a known recurrent PH, underwent a laparotomy for bowel obstruction where the PH was found unrelated to the obstruction but was reduced and the fascial orifice tightened. Notably, all three patients who underwent surgical repair for a recurrence following the LoPa procedure developed a subsequent re-recurrence. The fourth patient developed a stomal stricture which was surgically excised.

### Quality of Life

QoL questionnaires were completed by all but one who participated in follow-up. This was a patient who had a recurrence after the LoPa repair of an ileostomy but had a reversal of the stoma prior to the follow-up.

**FIGURE 3:**
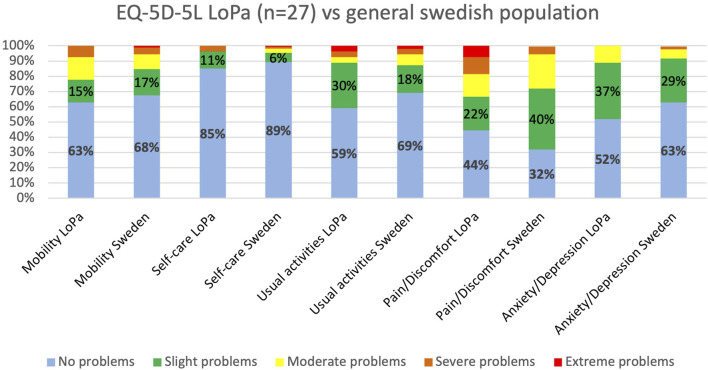
Comparison of EQ-5D-5L health profiles between the LoPa cohort and the general population. The chart displays the percentage of respondents reporting problems across the five health dimensions for patients after the LoPa procedure (n = 27) versus published reference values for the general Swedish population [31].

**FIGURE 4:**
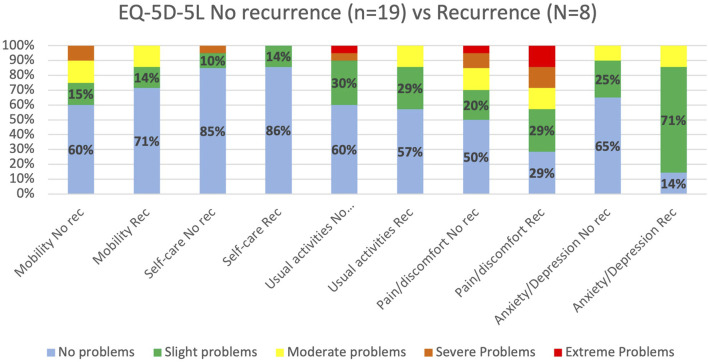
Comparison of EQ-5D-5L health profiles for LoPa patients with and without PH recurrence. The chart displays the percentage of patients reporting problems across the five health dimensions, comparing those who developed a recurrence after the LoPa procedure to those who did not (Total n = 27).

The overall health status after the LoPa procedure, according to the EQ-5D-5L, was comparable to the Swedish general population. LoPa patients reported a 12-percentage point higher incidence of slight problems with usual activities, whereas the general population reported an 18-percentage point higher incidence of slight problems with pain and discomfort, Figure 3. When comparing patients with and without recurrences after LoPa repair, minor differences in 3 dimensions were found. Patients with recurrence reported higher rates of severe and extreme pain/discomfort. In the ‘usual activity’ dimension, 14% of patients with a recurrence reported moderate problems, compared to 10% without recurrence reporting severe or extreme problems. Furthermore, patients with recurrence also reported increased rates of slight problems in the depression/anxiety dimension leading to a somewhat worse outcome for this dimension overall, see Figure 4.

**FIGURE 5:**
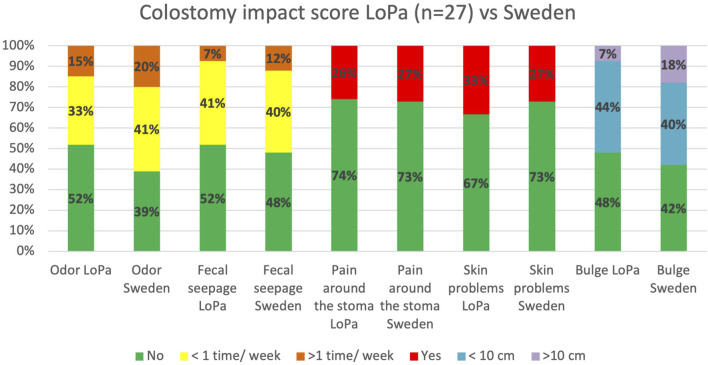
Comparison of selected items from the Colostomy Impact Score. The figure displays the percentage of patients in the LoPa cohort (n = 27) reporting specific stoma-related problems compared to a Swedish reference cohort [32]. To pragmatically accommodate all stoma types (colostomies, ileostomies, and ileal conduits), the “stool consistency” and “stoma care” items were excluded from this analysis.

**FIGURE 6:**
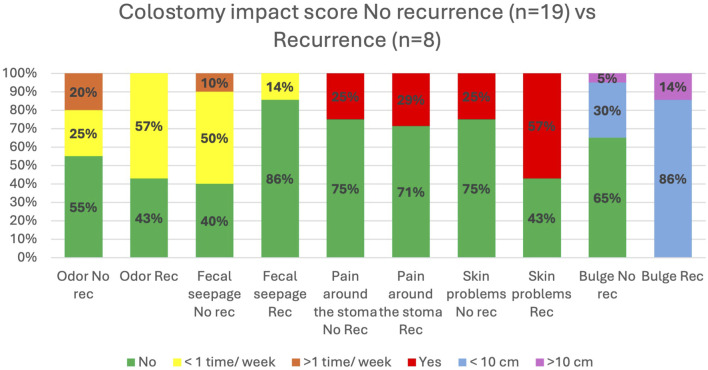
Comparison of stoma-related outcomes for LoPa patients with and without hernia recurrence. Based on selected items from the Colostomy Impact Score, the figure shows the percentage of patients reporting specific problems, comparing those who developed a recurrence after the LoPa procedure to those who did not (Total n = 27).

As for Colostomy Impact Score, the LoPa cohort reported 11 percentage points lower incidence of parastomal bulges larger than 10 cm, compared to the Swedish reference cohort Figure 5. LoPa patients with a recurrence reported more skin problems and bulging larger than 10 cm. On the other hand, patients without a recurrence reported a higher incidence of odour and faecal seepage, Figure 6.

## Discussion

This study demonstrates that LoPa is a safe and feasible method for repairing PHs with short LOS and few serious short-term complications. Over a median follow-up of 47 months, a recurrence was observed in 12 patients (33.3%), with 3 patients requiring reoperation for PH recurrence. The reported overall impact on QoL was relatively modest.

Recurrence remains a significant challenge in PH repair. With a relatively long median follow-up of 47 months, matched only by a few studies in the literature, our 33.3% recurrence rate is comparable to studies with similarly long observation periods (>24 months) [16–18, 34–36], while it appears high when compared against studies with shorter follow-up [22, 37–39]. This aligns with a general trend in the literature that recurrence rates after repair tend to increase with longer follow-up [1, 22, 40].

Diagnostic methods significantly influence reported rates of PH. Physical examination alone is unreliable for detecting small or asymptomatic cases [38, 41]. This contributes to the wide variability in reported recurrence rates, which are often lower in studies relying on physical examination for follow-up while reserving imaging for symptomatic or doubtful cases [14–21]. In our cohort, the availability of CT scans in 28 cases was invaluable, as it helped us identify recurrences when physical examination was not possible as well as one recurrence missed on physical exam. Consequently, the true recurrence rate is likely underestimated in studies that do not employ routine imaging for follow-up. This also applies to the 10 patients in our cohort for whom follow-up CT scans were unavailable.

Our cohort’s demographic, with an average age of 60–70 years and more than half of the patients classified as ASA 3-4, is comparable to other PH repair studies [14, 15, 23, 42–46]. This reflects a frail population with substantial comorbidities, a known risk factor for postoperative complications [47, 48]. The median operative time was relatively long, another established risk factor for complications [49]. While the operative time was comparable to other open repair methods [23, 44], it was longer than reported in laparoscopic repair studies [14, 38, 42, 45] The longer duration for the LoPa repair compared to laparoscopic repairs is likely due to the mandatory takedown and recreation of the stoma, and the sometimes cumbersome dissection and extensive suturing through a narrow skin opening.

The median LOS was 3 days (IQR 2–7). This aligns with some reports while being shorter than some other [14, 15, 23, 38, 42–46]. Discharge criteria were stringent with requirement of stomal passage due to the novelty of the procedure, potentially leading to longer LOS than necessary. Future early outpatient follow-up could enable even earlier discharge. This relatively short LOS in this aged and comorbid patient population likely reflects the limited surgical trauma associated with the LoPa technique.

A concern with the LoPa technique is the risk for contamination of the surgical site prior to stoma closure. The SSI and SSO rates observed in our study align with rates reported in other studies on open repair techniques [14, 23, 46], and suggests that the approach of initially closing the stoma orifice by suturing the mucosa prior to sterile draping is safe and effective. Despite the abovementioned risk factors, no complications classified as Clavien-Dindo ≥4 were observed.

PH reduces QoL and impose significant restrictions on daily life as shown by studies using the EQ-5D-5L and colostomy impact score [8, 9, 32]. In this context, our QoL findings after LoPa repair are noteworthy. First, the overall health status of all patients was comparable to that of the Swedish general population [31]. Second, using colostomy impact score, our cohort had an approximately 10 percentage point lower rate of large parastomal bulges (>10 cm) than the reference group of Swedish rectal cancer survivors [32]. While our study lacks preoperative data for a direct comparison on an individual level, these findings suggest that the LoPa procedure restores general QoL from a hernia-impaired level back to the population norm. Despite a recurrence in one-third of the patients, the QoL in the total cohort was not impaired. Only one-third of patients with a recurrence sought care for a suspected recurrence, and one-fourth required reoperation of recurrence, indicating a relatively modest impact of the recurrences. While these findings indicate a positive outcome from the repair, this interpretation must be made with caution.

Achieving a balanced mesh fit is an inherent challenge in any PH repair, as an overly tight fit risks obstruction or ischemia, while a loose fit increases the risk of recurrence [22, 50]. This challenge is underscored by the two patients in our cohort who were reoperated due to bowel obstruction and faecal impaction. While the LoPa technique allows for a customized mesh, we cannot determine if the recurrences in our study resulted from a suboptimal fit.

The management of patients with recurrent PH hernias remains challenging due to the lack of specific evidence-based guidelines for operative methods, and the high rates of re-recurrence and complications associated with surgical repair [51]. Our experience in 10 patients (6 with recurrent PH, 4 with prior prophylactic mesh) suggests that the LoPa repair is technically feasible in cases where a retromuscular mesh is already present. While no major short-term complications occurred, two recurrences were noted during long-term follow-up. However, the small sample size precludes firm conclusions about LoPa repair’s efficacy or safety in this sub-group of patients. Nevertheless, we believe the LoPa technique could be a valuable option in a tailored, algorithmic approach for managing both primary and recurrent PH.

This study has several important limitations. Primarily, the retrospective nature of data collection and the small number of patients, and the heterogeneity of stoma types prevents us from drawing more than cautious conclusions from this study. Furthermore, although all procedures were performed by experienced abdominal wall surgeons, the novelty of the LoPa technique means that a potential learning curve effect on the outcomes cannot be ruled out. Additionally, the fact that all procedures were performed at a designated centre for abdominal wall surgery may limit the generalizability of our results to less specialized centres. Finally, the absence of preoperative QoL data precluded a direct statistical analysis of postoperative improvement.

As a local retromuscular mesh repair, LoPa offers the advantages of avoiding a midline laparotomy, a benefit that can lead to a shorter length of stay and faster recovery. This approach also minimizes surgical trauma and the need for extensive adhesiolysis, thereby potentially reducing post-repair adhesions that could complicate future abdominal surgeries. Conceptually, it is a modified retromuscular keyhole repair featuring a mesh collar that curves upwards along the stoma bowel, protecting it from the sharp edges of a flat mesh. While this design increases the mesh surface area in contact with the bowel to theoretically provide superior support, it did not prevent a recurrence rate of approximately one-third in our study.

## Conclusion

The LoPa technique is safe and feasible for repairing PH in patients without a symptomatic concomitant incisional hernia. It is characterized by low short-term morbidity and limited surgical trauma, making it an option especially for patients with significant comorbidities. While long-term recurrence rates are high, they are comparable to other methods. These preliminary findings require validation in larger, prospective trials to define the procedure’s definitive role in the surgical management of PH.

## Data Availability

The original contributions presented in the study are included in the article/supplementary material, further inquiries can be directed to the corresponding author.
